# Functional Trait Responses of *Sophora alopecuroides* L. Seedlings to Diverse Environmental Stresses in the Desert Steppe of Ningxia, China

**DOI:** 10.3390/plants13010069

**Published:** 2023-12-25

**Authors:** Jingdong Zhao, Chaoyi Shi, Le Wang, Xuejiao Han, Yuanjun Zhu, Jiankang Liu, Xiaohui Yang

**Affiliations:** 1 Breeding Base for State Key Lab. of Land Degradation and Ecological Restoration in Northwestern China/Key Lab. of Restoration and Reconstruction of Degraded Ecosystems in Northwestern China of Ministry of Education, Ningxia University, Yinchuan 750021, China; 2 Institute of Desertification Studies, Chinese Academy of Forestry, Beijing 100091, China; 3 Institute of Ecological Conservation and Restoration, Chinese Academy of Forestry, Beijing 100091, China; 4 Inner Mongolia Water Resources Inner Mongolia Water Resources Co., Ltd., Hohhot 010020, China; 5 Forestry and Grassland Work Station of Inner Mongolia, Hohhot 010011, China

**Keywords:** sand burial, salinity, drought, growth, *Sophora alopecuroides* L.

## Abstract

The seedling stage of plants is a crucial and vulnerable period in population and community dynamics. Despite this, studies on how plant traits respond to different environmental stresses often tend to overlook this early stage. Our study focused on *Sophora alopecuroides* L. seedlings in Ningxia Yanchi desert steppe, analyzing the effects of sand burial, salinity, and drought on their key aboveground and belowground traits. The results showed that sand burial significantly negatively affected stem biomass (SB), leaf biomass (LB), stem diameter (SD), leaf length (LL), leaf width (LW), leaf area (LA), and total root volume (RV), but positively influenced total root length (RL). As sand burial depth increased, SB, LB, SD, LL, LW, LA, RV, root biomass (RB), RV, and lateral root numbers (LRN) significantly decreased. Salinity stress negatively affected SB, LB, SD, LL, LW, LA, RB, RL, and RV, with these traits declining as the stress concentration increased. Drought stress had a positive effect on SD and LL, with both traits showing an increase as the intensity of the drought stress intensified; however, it adversely affected RL. In Ningxia Yanchi desert steppe, salinity stress had the most significant effect on the traits of *S. alopecuroides* seedlings, followed by sand burial, with drought having the least significant effect. This study provides essential theoretical support for understanding how *S. alopecuroides* seedlings cope with environmental stresses in their early life stages.

## 1. Introduction

As one of the fragile steppe ecosystems, the desert steppe’s biodiversity and ecological stability are facing dual challenges from biotic and abiotic stresses [1]. Biotic stress mainly involves grazing animals and human activities, such as grazing, trampling, mowing, and cultivation, while abiotic stress primarily relates to environmental and climatic factors, often beyond direct human control [2]. In the wild, plants must adapt to these prolonged and extreme climatic conditions to survive [3,4,5]. Wind erosion, characteristic of semi-arid and arid regions, manifests as thick layers of sand covering the soil surface [6,7], not only threatening seed germination but also challenging the growth and development of seedlings [8]. Moreover, due to the arid climate, scarce precipitation, and intense evaporation in the desert areas of northwest China, extensive saline soils have formed, and this phenomenon leads to soil salinity accumulation and frequent droughts [2], further exacerbating the survival challenges for plants. Previous studies have found that research on plant functional traits can help us to better understand, quantify, and simulate key ecological processes [9], particularly the responses of species and populations to abiotic stresses [10].

The early stages of seedling growth represent the most vulnerable phase in a plant’s life cycle [11]. The various organs of the seedling, during these initial stages of growth, offer ideal materials for studying how plants adapt to their environment [12]. Changes in leaf size (leaf area, leaf length, leaf width) are critical indicators of plant responses to light and water stress. For instance, a larger leaf area can enhance the area available for photosynthesis, while smaller leaves can reduce water evaporation, thereby improving water use efficiency, a common adaptation in environments with scarce rainfall [13]. The stem diameter reveals adaptations in mechanical strength and water transport capabilities, crucial for plant stability under wind or other physical pressures [14]. Root traits, such as root biomass, total root length, and total root volume, are key factors influencing soil nutrient dynamics and fertility [15], indicating plant adaptability in various soil types and nutritional conditions. Rapid root growth is essential for plant establishment in arid systems [12]. Studying these traits helps to deepen our understanding of how plants adapt their growth forms to ever-changing environmental conditions, thereby aiding in predicting and addressing climate change and other environmental stresses.

*Sophora alopecuroides* L., an herbaceous plant in the legume family, is widely distributed in the deserts and semi-deserts of northern China. It is often used to prevent land desertification and reduce soil erosion due to its resistance to wind, saline–alkali conditions, and drought [16,17]. However, the desert steppes are becoming increasingly fragile due to the interaction of biotic and abiotic factors, and we still know little about the growth and adaptation mechanisms of *S. alopecuroides* seedlings under complex environmental stresses. Previous trait-based frameworks often overlook this early life stage of plants [18]. A comprehensive understanding and study of plant seedlings’ responses to environmental stress are crucial for predicting and managing population and community dynamics. In light of this, our study uses *S. alopecuroides* seedlings as experimental material. By analyzing the specific effects of three common stresses in desert steppes on the aboveground and underground traits of *S. alopecuroides* seedlings, this research explores how different components of the seedlings regulate themselves to cope with environmental stress, providing key information for predicting and managing the response of *S. alopecuroides* seedlings to environmental changes. Specifically, we proposed two hypotheses. First, similar to the variability in traits during the adult phase of plants [19], we believed that plant traits also underwent specific changes during the seedling stage under different stress conditions. Second, due to the varying tolerance levels of *S. alopecuroides* seeds during germination to different stresses [20], we believed that the intensity of the effect of different stresses on the traits of *S. alopecuroides* seedlings also varied.

## 2. Results

### 2.1. Factors Affecting the Aboveground Traits of S. alopecuroides Seedlings

**Figure 1:**
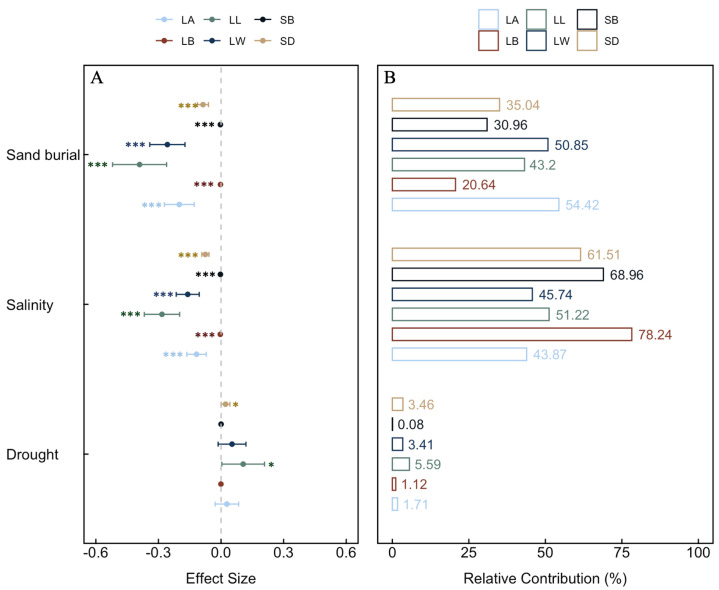
The effect size and relative contribution of different stresses on the aboveground traits of *S. alopecuroides* seedlings. (**A**): Effect size, (**B**): Relative contribution. SB, LB, SD, LL, LW, and LA, respectively, represent stem biomass, leaf biomass, stem diameter, leaf length, leaf width, and leaf area. Significance marks: ***: *p* < 0. 001; *: *p* < 0.05; left of error bars for negative effects, right for positive effects.

During the growth of *S. alopecuroides* seedlings, sand burial, and salinity significantly negatively affected all aboveground traits (*p* < 0.001), while drought positively influenced all aboveground traits except leaf biomass (LB), with significant effects on stem diameter (SD) and leaf length (LL) (*p* < 0.05) (Figure 1A). Compared to drought, sand burial and salinity had a greater effect on the aboveground traits. Sand burial had the largest effect on leaf width (LW) (50.85%) and leaf area (LA) (54.42%), while salinity had the greatest effect on SD (61.51%), LL (51.22%), LB (78.24%), and stem biomass (SB) (68.96%) (Figure 1B).

### 2.2. Factors Affecting the Belowground Traits of S. alopecuroides Seedlings

**Figure 2:**
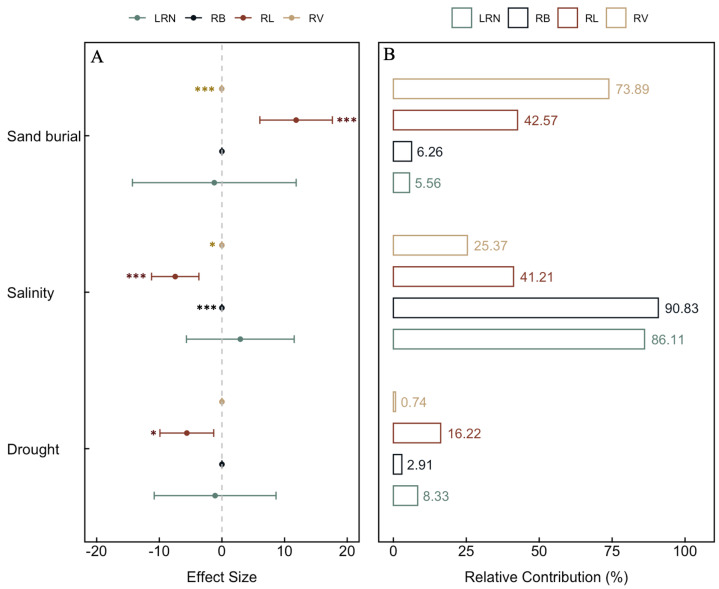
The effect size and relative contribution of different stresses on the belowground traits of *S. alopecuroides* seedlings. (**A**): Effect size, (**B**): Relative contribution. RB, RL, RV, and LRN, respectively, represent root biomass, total root length, total root volume, and lateral root numbers. Significance marks: ***: *p* < 0. 001; *: *p* < 0.05; left of error bars for negative effects, right for positive effects.

During the growth of *S. alopecuroides* seedlings, sand burial significantly positively affected total root length (RL) (*p* < 0.001) and negatively affected total root volume (RV) (*p* < 0.001), but had non-significant negative effects on root biomass (RB) and lateral root numbers (LRN); salinity had significant negative effects on RB (*p* < 0.001), RL (*p* < 0.001), and RV (*p* < 0.05), and a non-significant positive effect on LRN; and drought negatively affected all belowground trait indicators, but only significantly affected RL (*p* < 0.05) (Figure 2A). According to the relative contributions of each predictor, sand burial had the greatest effect on RV (73.89%) and RL (42.57%); salinity had the greatest effect on RB (90.83%) and LRN (86.11%); and the effect of drought on all belowground trait indicators was relatively small (Figure 2B).

### 2.3. Relationship between Different Stresses and Traits in S. alopecuroides Seedlings

**Figure 3:**
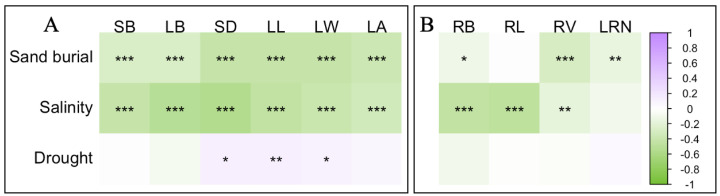
Pearson correlations between different stresses and aboveground (**A**); and belowground (**B**) traits in *S. alopecuroides* seedlings. SB, LB, SD, LL, LW, LA, RB, RL, RV, and LRN, respectively, represent stem biomass, leaf biomass, stem diameter, leaf length, leaf width, leaf area, root biomass, total root length, total root volume, and lateral root numbers. Significance marks: ***: *p* < 0. 001; **: *p* < 0.01; *: *p* < 0.05.

The correlation between different stresses and both aboveground and belowground traits in *S. alopecuroides* seedlings indicated that the negative effects of various stresses on traits were more prevalent than positive effects, with positive effects mainly observed under drought stress (Figure 3). In aboveground traits, sand burial and salinity stress showed a significant negative correlation with SB, LB, SD, LL, LW, and LA, while drought stress was significantly positively correlated with SD, LL, LW, and showed no significant correlation with SB, LB, and LA (Figure 3A). In belowground traits, sand burial stress exhibited a significant negative correlation with RB, RV, and LRN, and no significant correlation with RL; salinity stress was significantly negatively correlated with RB, RL, RV, and showed no significant correlation with LRN; however, drought stress did not show any significant correlation with the four belowground traits (Figure 3B). Overall, the aboveground traits of *S. alopecuroides* seedlings responded more strongly to stresses than belowground traits, with the effect of sand burial and salinity stress being notably greater than that of drought stress.

**Figure 4:**
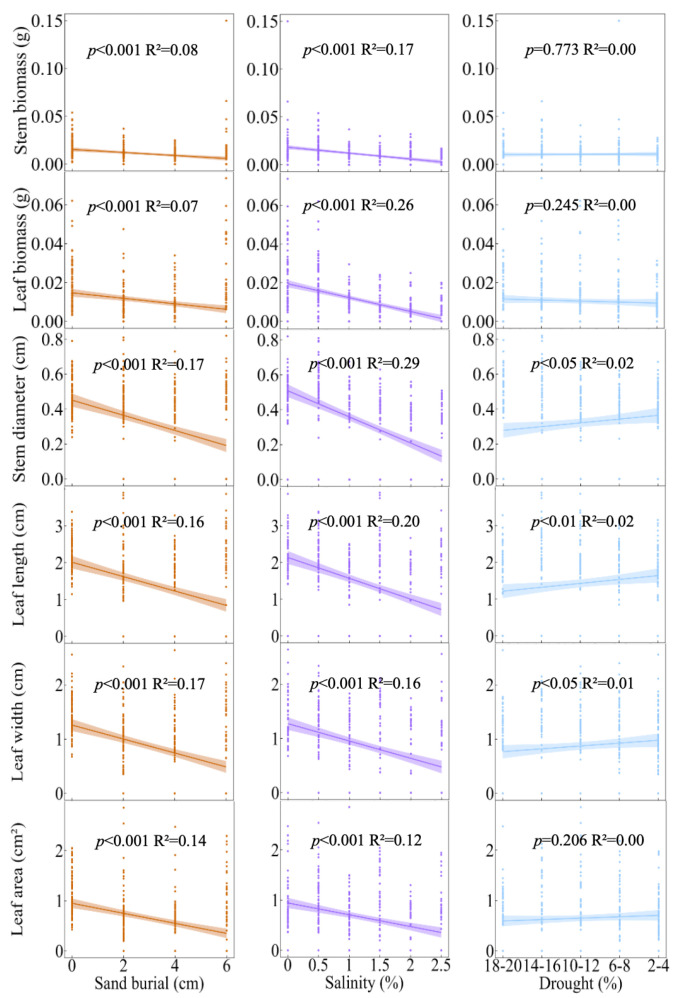
Relationships between different stresses and aboveground traits in *S. alopecuroides* seedlings. The shaded area shows the 95% confidence interval of the regression. Sand burial (sand depth): 0 cm, 2 cm, 4 cm, 6 cm; salinity (soil saline–alkali content): 0%, 0.5%, 1%, 1.5%, 2%, 2.5%; and drought (soil moisture content): 18–20%, 14–16%, 10–12%, 6–8%, 2–4%.

**Figure 5:**
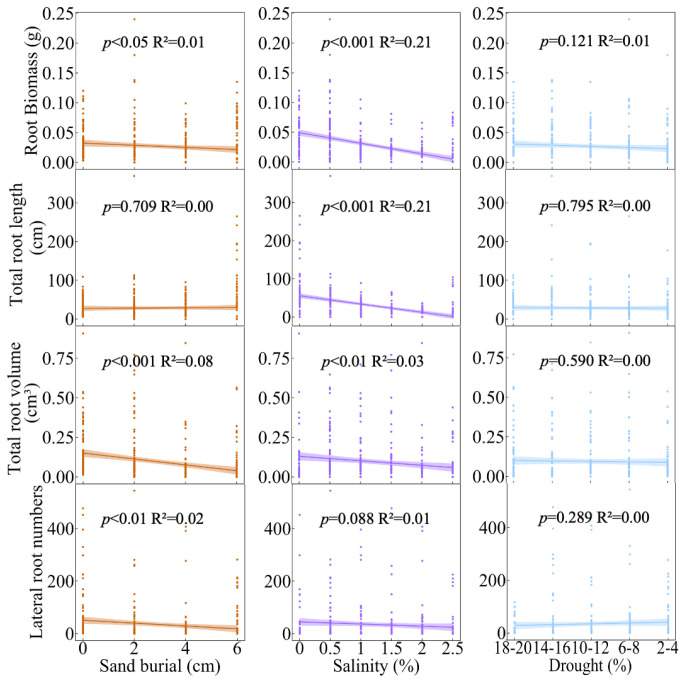
Relationships between different stresses and belowground traits in *S. alopecuroides* seedlings. The shaded area shows the 95% confidence interval of the regression. Sand burial (sand depth): 0 cm, 2 cm, 4 cm, 6 cm; salinity (soil saline–alkali content): 0%, 0.5%, 1%, 1.5%, 2%, 2.5%; and drought (soil moisture content): 18–20%, 14–16%, 10–12%, 6–8%, 2–4%.

In the aboveground traits of *S. alopecuroides* seedlings, with increasing sand burial, SB, LB, SD, LL, LW, and LA all exhibited a significant decreasing trend (R^2^_SB_ = 0.08, *p* < 0.001; R^2^_LB_ = 0.07, *p* < 0.001; R^2^_SD_ = 0.17, *p* < 0.001; R^2^_LL_ = 0.16, *p* < 0.001; R^2^_LW_ = 0.17, *p* < 0.001; R^2^_LA_ = 0.14, *p* < 0.001). Similarly, with increasing salinity, the aboveground traits of *S. alopecuroides* seedlings, including SB, LB, SD, LL, LW, and LA, also showed a significant decreasing trend (R^2^_SB_ = 0.17, *p* < 0.001; R^2^_LB_ = 0.26, *p* < 0.001; R^2^_SD_ = 0.29, *p* < 0.001; R^2^_LL_ = 0.20, *p* < 0.001; R^2^_LW_ = 0.16, *p* < 0.001; R^2^_LA_ = 0.12, *p* < 0.001). Conversely, as drought severity increased, SD, LL, and LW showed a significant increasing trend (R^2^_SD_ = 0.02, *p* < 0.05; R^2^_LL_ = 0.02, *p* < 0.01; R^2^_LW_ = 0.01, *p* < 0.05), while changes in SB, LB, and LA were not significant (Figure 4). In the belowground traits of *S. alopecuroides* seedlings, with increasing sand burial, RB, RV, and LRN showed a significant decreasing trend (R^2^_RB_ = 0.01, *p* < 0.05; R^2^_RV_ = 0.08, *p* < 0.001; R^2^_LRN_ = 0.02, *p* < 0.01), while RL changes were not significant. With increasing salinity, RB, RL, and RV exhibited a significant decreasing trend (R^2^_RB_ = 0.21, *p* < 0.001; R^2^_RL_ = 0.21, *p* < 0.001; R^2^_RV_ = 0.03, *p* < 0.01), while LRN changes were not significant. As drought severity increased, changes in RB, RL, RV, and LRN were all not significant (Figure 5).

## 3. Discussion

In arid and semi-arid environments, poor seed germination and seedling establishment are major issues, and these traits are considered crucial factors affecting later plant growth and yield [21]. Researchers have noted that the environmental context is a key element in ecological studies, and when environmental stress factors become too extreme, situations may arise where no seedlings can survive, regardless of their ecological traits [18]. The latest research on the germination characteristics of *S. alopecuroides* seeds under different stress conditions found that sand burial is the most critical stress limiting seed germination [20]. A thick layer of sand can induce seed dormancy; however, even if seeds are successfully activated, seedlings might fail to germinate due to an inability to break through the sand layer [22]. Moreover, studies have found that the overall effect on plants is minimal when each individual stress is applied to seedlings. However, as the combination and complexity of multi-factor stresses increase, plant survival rate, root growth, and chlorophyll content all decline [5]. In this study, some *S. alopecuroides* seedling trait values were zero, indicating that certain stress combinations indeed restricted seedling growth. To accurately handle these zero values, we employed a zero-inflated Gaussian mixture model for data processing. This model allowed us to consider the scenarios of plants not growing at all and actual growth. The results showed that different stresses had varying directions and intensities of effect on the aboveground and underground traits of *S. alopecuroides* seedlings, with negative effects generally being more prevalent than positive ones. Notably, the effects of sand burial and salinity stress were much greater than those of drought stress. These results therefore support our two hypotheses.

The aboveground parts of plant seedlings, primarily consisting of stems and leaves, play a crucial role in their growth and adaptation. The leaves are the main sites of photosynthesis [13], while stems not only support the overall structure of the plant but also transport nutrients, water, and photosynthetic products [23]. Therefore, studying the adaptive functional traits of the aboveground parts of seedlings is vital for understanding plant growth patterns, photosynthetic efficiency, and environmental adaptability. This study found that sand burial and salinity stress generally have negative effects on the aboveground parts of plants, with different traits showing significant declines as the stress gradients increase. Previous research has identified sand burial as a primary stressor affecting the growth, survival, and distribution of sand dune plants [8]. Compared to conditions without sand burial, plants under sand burial must invest more in the stems that are buried in the sand layer, and this investment tends to increase with the depth of sand burial. Consequently, seedlings need to reduce their SD to penetrate the sand layer, while also decreasing LL, LW, and LA to lower energy expenditure. Salinity and alkalinity are two coexisting abiotic stresses. Combined saline–alkali stress causes osmotic, oxidative, and ionic toxicity stresses and high pH stress, which can disrupt the cell membrane structure of plants, deactivate enzyme activities, and disrupt the ion balance within cells [24,25], significantly affecting the accumulation of plant biomass [26]. Interestingly, in this study, drought had a positive effect on SD and LL. Previous studies have shown that the overproduction of reactive oxygen species is a key mechanism behind plant responses to water stress, which inhibits seed germination and seedling growth [27]. Additionally, in semi-arid or desert climates, plant leaves are generally smaller and narrower to minimize water loss [28]. However, this study found different results. The SD, LL, and LW of *S. alopecuroides* seedlings were significantly positively correlated with drought stress (Figure 3) and increased with the intensification of drought (Figure 4), indicating the better adaptability of *S. alopecuroides* seedlings to drought conditions. When facing insufficient environmental water, the seedlings increase their investment in the stem and leaf parts to ensure normal photosynthesis.

Plants absorb the necessary water and minerals from the soil through their roots to support their growth and development. The proliferation of lateral roots enhances the distribution range of the root system, and the structure of the roots largely depends on the repeated formation of new lateral roots [29]. This study found that, unlike the traits of the aboveground parts, sand burial has a significant positive effect on the RL of *S. alopecuroides* seedlings, but a significant negative effect on RV (Figure 2A). This could be due to physiological and morphological adjustments made by *S. alopecuroides* seedlings to adapt to the sand burial environment. These adjustments include increasing the RL to explore a larger range of water and nutrients, while reducing the RV under limited resource conditions to save energy. Salinity conditions have a significant negative effect on RB, RL, and RV. When plants are subjected to salinity stress, the roots are the first organ to perceive the stress, which is then gradually transmitted to the aboveground parts. The normal growth, development, and physiological and biochemical metabolism of the plant are severely disrupted [24]. Our study also found that salinity stress contributed up to 90.83% and 86.11% to RB and LRN, respectively (Figure 2B), while in aboveground traits, the maximum contribution of salinity was mainly reflected in LB, at only 78.24% (Figure 1B). This indicates that the root system is, indeed, the first organ affected by salinity stress. In contrast to the aboveground traits, drought has a negative effect on all underground traits, with a significant effect on RL, suggesting that, under drought stress, *S. alopecuroides* seedlings prioritize resource allocation to the aboveground parts, reflecting the plant’s resource distribution strategy in response to stress.

A comprehensive analysis of the effect of various stresses on the aboveground and underground traits of *S. alopecuroides* seedlings revealed that salinity stress had the greatest effect on SD (61.51%), LL (51.22%), LB (78.24%), SB (68.96%), RB (90.83%), and LRN (86.11%) (Figure 1B and Figure 2B). This suggests that high salt concentrations, causing osmotic changes and ionic toxicity, not only restrict water and nutrient absorption but also trigger a range of physiological stress responses. This combination adversely affects the overall growth and development of the seedlings. Soil salinization is a critical issue affecting seedling growth in many regions [30]. Plant survival under alkaline conditions depends on their ability to cope with water stress and ionic toxicity, as well as their resistance to high pH levels. Therefore, compared to individual salt stress, plants expend more energy and resources to adapt to combined saline–alkali stress [31]. Conversely, drought stress had the least effect on these traits, indicating that *S. alopecuroides* seedlings might have better adaptability to arid environments. Furthermore, from some perspectives, salt and drought stresses are physiologically related, as both induce osmotic stress, and most metabolic responses in affected plants are somewhat similar [32]. This could also be due to the greater pressure exerted by saline–alkali stress on seedlings, thus weakening the negative effects of drought. As plant water regulation is highly complex, involving the coordination of many traits and feedback with environmental conditions, further research is needed in this area [10].

## 4. Materials and Methods

### 4.1. Study Area

**Figure 6:**
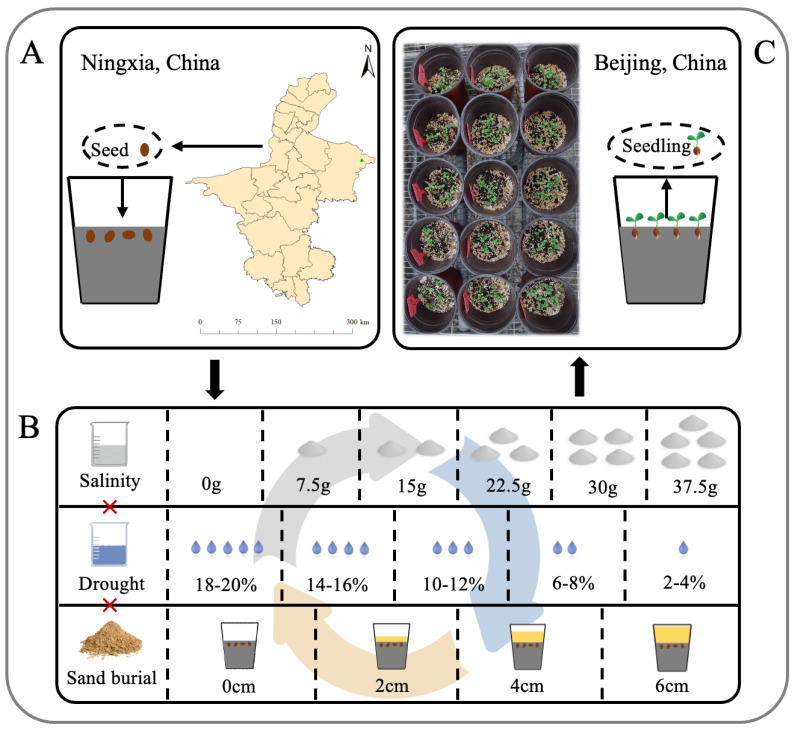
Experimental design diagram. (**A**): *S. alopecuroides* seeds sampling location (the green triangle symbol), (**B**): Stress treatment, (**C**): Greenhouse cultivation. Sand burial (sand depth): 0 cm, 2 cm, 4 cm, 6 cm; salinity (soil saline–alkali content): 0%, 0.5%, 1%, 1.5%, 2%, 2.5%; and drought (soil moisture content): 18–20%, 14–16%, 10–12%, 6–8%, 2–4%.

This study was conducted in a greenhouse at the Institute of Desertification Studies, Chinese Academy of Forestry, with average day/night temperatures approximately maintained at 25–32 °C and 15–23 °C, air humidity at approximately about 30–40%, and an average photoperiod of approximately 13 h. We sourced mature *S. alopecuroides* seeds from the indigenous flora of Yanchi County, Ningxia Autonomous Region, China (37°50′07.516″ N, 107°25′23.873″ E), harvested in September 2021 (Figure 6A,C). This region is characterized by its unique climatic and soil conditions: an average annual rainfall of approximately 285.6 mm; an average temperature of 8.2 °C; and persistent dryness throughout the year coupled with high evaporation rates and frequent aeolian sand activity. The prevalent sierozem soil in this area is notably low in organic matter and nutrients. The native vegetation primarily consists of drought-resistant xerophytic herbs, semi-shrubs, and shrubs. Dominant among these are *S. alopecuroides*, *Astragalus melilotoides*, and *Stipa caucasica* subsp. *glareosa* [20,33]. Our experimental setup aimed to closely mimic these natural conditions, providing valuable insights into the adaptive mechanisms of *S. alopecuroides* in its native habitat.

### 4.2. Experimental Design

The specific methods for the pre-treatment of *S. alopecuroides* seeds before germination can be found in the paper published by Zhao et al. [20]. In June 2022, we initiated our experiment with the preparation of *S. alopecuroides* seeds, using plastic flowerpots measuring 16.5 cm in height and 13.5 cm in diameter as planting containers. Each pot was filled with about 1.5 kg of a 4:1 volume ratio mixture of nutritive soil and perlite, which served as the initial medium for all treatments. After planting 30 healthy seeds in each pot, they were lightly covered with a thin layer of soil. Our experimental design (Figure 6B) encompassed a range of sand burial, salinity, and drought stress conditions. The sand burial stress was applied in 4 distinct levels: 0 cm (no burial), 2 cm, 4 cm, and 6 cm, using sand sourced from the Yanchi desert steppe. The salinity stress was divided into 6 levels, achieved by adding saline–alkali solutions with varying quantities of mixed salts (0 g, 7.5 g, 15 g, 22.5 g, 30 g, and 37.5 g) to the soil. These solutions contained NaCl, Na_2_SO_4_, and NaHCO_3_ in a 1:2:1 molar ratio, resulting in soil saline–alkali contents of 0%, 0.5%, 1.0%, 1.5%, 2.0%, and 2.5%. Drought stress was implemented in 5 stages, with soil water content levels set at 18–20%, 14–16%, 10–12%, 6–8%, and 2–4%, respectively. Upon reaching the target soil saline–alkali content for each treatment, the pots were watered with fresh water, and soil moisture levels were carefully maintained using a weighing method. The applied levels of salinity and drought stress were designed based on data from previous similar studies conducted in the same region [34,35]. A total of 120 multifactor stress treatments were tested, with each treatment replicated 3 times to ensure comprehensive and reliable data. Due to the differences in stress intensity under different treatments, 21 days after seed sowing, when germination ended, if the number of surviving seedlings in the flowerpot was 5 or more, 5 seedlings were established per pot. If there were fewer than 5 seedlings, all were retained. Above- and belowground biomass and morphological traits of the plants were measured 3 months after the formal treatment.

### 4.3. Data Collection and Analysis

First, the aboveground and underground parts of the *S. alopecuroides* seedlings were separated using scissors. The SD at the junction of the root and stem was measured using an electronic caliper (precision of 0.01 mm). Healthy leaves were laid flat on coordinate paper (17 × 25cm), and the roots were thoroughly washed with clean water and then spread out on a white hardboard. Subsequently, a digital scanner was used to scan both the leaves and the root system separately. The LL, LW, and LA were measured using ImageJ2 v2.3.0 software (National Institutes of Health, Bethesda, MD, USA). RL, RV, and LRN were obtained through the analysis of root system images using root analysis software (WinRHIZO Pro v2.0). Finally, the aboveground part of the plant was kill-greened at 105 °C for 15 min and then dried at 80 °C to a constant weight. The root system was dried at 65 °C to a constant weight. The SB, LB, and RB of the plant were measured using a milligram balance. In this study, SB, LB, SD, LL, LW, and LA were considered as the aboveground traits of *S. alopecuroides* seedlings, and RB, RL, RV, and LRN as the belowground traits.

In this study, we focused on the growth characteristics of plants under different environmental stress conditions. Observational data showed that in some cases, plants failed to exhibit the expected growth traits, leading to a large number of zero values. These zero values are not due to data missing or recording errors but are a true reflection of the biological phenomenon where plants do not grow under certain environmental stresses. Traditional statistical models often fail to properly handle this type of data, as they do not take into account the special nature and generation mechanism of zero values. Zero-inflated models provide us with a statistically sound and biologically meaningful method. Moreover, the plant growth data collected in this study are continuous, not integer data. Many traditional zero-inflated models, like the zero-inflated Poisson model, are usually used for count data [36]. Therefore, we used a zero-inflated Gaussian mixture model to handle the non-zero continuous data. This model combines the characteristics of zero-inflated and Gaussian mixture models, effectively modeling both zero and non-zero values [37]. In the zero-inflated part, the model assesses the probability of zero values occurring, reflecting situations where plants do not grow under certain environmental conditions. The Gaussian distribution part is responsible for modeling non-zero data, i.e., the actual growth of the plant. This model design allowed us to more accurately understand and predict changes in plant growth traits under different environmental stresses. Additionally, the effect sizes were calculated through coefficient estimates. These estimates reflected the average effect of each stressor on the response variables. The relative contributions were calculated using hierarchical partitioning analysis, thereby determining the relative importance of each stressor [38]. Finally, to further understand the relationships between various traits and different stress conditions, this study employed linear fitting to explore their linear relationships. We primarily conduct zero-inflated model fitting using the glmmTMB v 1.1.8 package in R [39] and utilize the ggplot2 v3.4.4 package for data visualization [40].

## 5. Conclusions

The variation in seedling traits under different environmental stresses provides insights into plant adaptability during early growth stages. In this study, sand burial and salinity stresses had a significantly greater effect compared to drought on both aboveground and underground traits of *S. alopecuroides* seedlings. Sand burial had a significant negative effect on SB, LB, SD, LL, LW, LA, and RV, but a positive effect on RL. Except for LRN, salinity stress negatively affected all other indicators. Drought stress positively affected SD and LL, but negatively influenced RL. As sand burial depth increased, all indicators except RL showed a significant decreasing trend. With increasing saline–alkali concentration, all indicators except LRN demonstrated a marked decline. Under increasing drought conditions, only SD, LL, and LW showed a significant increasing trend. This study reveals that, compared to sand burial and salinity stress, *S. alopecuroides* seedlings are more adaptable to drought. The enduring and intense stress of sand burial and salinity environments necessitates more significant physiological and morphological adjustments in plants for adaptation. The severity of different stresses is ranked as salinity > sand burial > drought. This research highlights how seedlings adapt to various environmental stresses from the perspective of functional traits in their early growth stages.

## Data Availability

Data are contained within the article.
